# Microcontroller Implementation of LSTM Neural Networks for Dynamic Hand Gesture Recognition

**DOI:** 10.3390/s25123831

**Published:** 2025-06-19

**Authors:** Kevin Di Leo, Giorgio Biagetti, Laura Falaschetti, Paolo Crippa

**Affiliations:** DII—Dipartimento di Ingegneria dell’Informazione, Università Politecnica delle Marche, Via Brecce Bianche 12, I-60131 Ancona, Italy; S1116240@studenti.univpm.it (K.D.L.); g.biagetti@univpm.it (G.B.); l.falaschetti@univpm.it (L.F.)

**Keywords:** LSTM, neural networks, hand gesture recognition, STM32, microcontroller, embedded systems, accelerometer

## Abstract

Accelerometers are nowadays included in almost any portable or mobile device, including smartphones, smartwatches, wrist-bands, and even smart rings. The data collected from them is therefore an ideal candidate to tackle human motion recognition, as it can easily and unobtrusively be acquired. In this work we analyze the performance of a hand-gesture classification system implemented using LSTM neural networks on a resource-constrained microcontroller platform, which required trade-offs between network accuracy and resource utilization. Using a publicly available dataset, which includes data for 20 different hand gestures recorded from 10 subjects using a wrist-worn device with a 3-axial accelerometer, we achieved nearly 90.25% accuracy while running the model on an STM32L4-series microcontroller, with an inference time of 418 ms for 4 s sequences, corresponding to an average CPU usage of about 10% for the recognition task.

## 1. Introduction

The huge amount of data collected by modern commercial wearable devices, thanks to their advanced sensing capabilities, has made it necessary to perform data analysis directly on board these devices in order to increase their energy efficiency, reducing the amount of data sent via RF links (e.g., WiFi, Bluetooth Low Energy (BLE), ZigBee, …). In this sense, the wearable device can be considered the “edge” to a mobile phone, a central station, or a web server, which traditionally would perform more complex data processing [1].

Indeed, the progress of microelectronics has allowed integrated circuit manufacturers to increase the computational capabilities of their microcontrollers used in today’s mobile and wearable devices. This has consequently allowed the porting onto microcontrollers of various machine learning tools for pattern recognition, classification, and prediction, such as recurrent neural networks (RNNs) or long short-term memory (LSTM) neural networks (NNs) [2,3,4,5,6,7,8,9]. However, trade-offs between network accuracy and resource utilization should be carefully considered in firmware implementations on low-power and low-cost wearable microcontroller platforms, where computational resource constraints severely impact the overall design of the sensor system [10].

To facilitate the embedding of machine learning algorithms into wearable devices, there is a wide range of frameworks such as MicroPython and Lite Runtime (LiteRT), formerly known as TensorFlow Lite (TFLite) for Microcontrollers, that provide a set of tools to run neural network models on supported microcontrollers for pattern recognition and classification.

The TensorFlow platform [11] makes it easy to build machine learning (ML) models that run in any environment and can be used for a variety of ML tasks, such as image and text classification, time series forecasting, and pattern recognition. One of the most useful high-level frameworks for using the TensorFlow platform is Keras, which lets designers create neural network models by specifying layer types and sizes, and automatically performs fitting and evaluation of the models. This greatly simplifies the process of designing and training neural networks, allowing for rapid testing, modification, and  optimization. In this direction, STMicroelectronics has achieved progress in research on the implementation of ML and deep learning (DL) models using the Keras, TFLite or PyTorch frameworks on memory-limited STM32 microcontrollers [12], supported by the X-CUBE-AI extension package [13].

Since mobile and wearable devices have proven to be very useful for human activity recognition (HAR) [10,14,15,16], in this work we considered specific branches of HAR such as sign language recognition [17,18,19] or dynamic hand gesture detection and classification [20,21] as simple and common interesting application scenarios for wearable devices. Gesture recognition requires a model or classification scheme whose complexity depends on the number of output classes needed. Examples include using an acceleration-based wearable device as a human–computer interface, such as for text input, drawing numbers and letters, sending commands by moving fingers and hands in the air, or  determining what the user is doing at a given moment. These are multiclass classification tasks that are an excellent use case for NNs. Traditional multiclass methods for gesture recognition, such as extended support vector machines (SVMs) and k-nearest neighbors (kNNs), can offer good accuracy at the expense of the need for complex models [22]. It is well documented that using LSTM networks for human activity classification [23,24] has a high degree of accuracy. As a result, LSTM neural networks, which include a memory element in each neuron, are well suited to efficiently model time series such as acceleration-based dynamic gesture recognition. For example, in [25], DL methods using TensorFlow and Keras were evaluated in gesture recognition. A multi-layer LSTM model was trained and evaluated on data (10 gestures, 1000 trials total) from a finger ring device collecting acceleration data, achieving a per-gesture accuracy ranging from 75 to 95%, but the model turned out to be too big and/or complex to be implemented on the edge device.

The major disadvantage of larger, highly accurate networks is indeed their size, as they cannot be easily stored and loaded onto embedded devices, due to memory limitations. This can be mitigated by fusing operations in a network together, at the cost of accuracy. This is how the TFLite converter utility functions and can be used to turn Keras models into microcontroller-compatible compressed TFLite models.

Training a neural network can be a very computationally expensive task, which can be shortened by using hardware accelerators such as a graphics processing unit (GPU) or a tensor processing unit (TPU). The easiest and most straightforward way to leverage these accelerators is to use the free, cloud-based ML platform Google Colaboratory (“Colab”): Colab offers a Jupyter Notebook along with a Python environment with scikit-learn, TensorFlow, Keras, PyTorch, and other libraries designed for ML.

In this work, Colab was used to generate models for STM32CubeIDE 1.16.0 with the X-CUBE-AI 10.0 tool to implement the LSTM neural network for a hand gesture classification system on an STMicroelectronics STM32L4-series microcontroller. The novelty of this paper lies in the definition of an NN architecture optimized to fit the available 1 MiB of memory embedded in the selected microcontroller, an STM32L476RG, chosen for its low-power architecture that makes it ideal to be used in the design of a wearable smart sensor. This has been achieved through the derivation of an analytical model of network complexity (memory usage and computational cost) as a function of design variables (number of neurons and layer types) and extensive experimentation to select the best compromise between accuracy and network size. The good performance of the classification system was finally verified by running the model on the real microcontroller using the publicly available “SmartWatch Gesture Dataset” [26], which includes data for 20 different hand gestures recorded by 10 subjects using a wrist-worn device with a triaxial accelerometer. A 4-fold subject-based cross-validation showed that the NN achieved an average accuracy of 90.25%.

The main contribution of this paper is the design of a neural network-based gesture recognizer constrained to run on the limited resources available on a wearable embedded system. To this end, this paper presents (i) a detailed statistical analysis of the gesture durations to minimize the length of the data vectors to be processed by the neural network, (ii) an accurate analysis of the memory consumption and computational complexity of LSTM-based neural networks that allowed us to select a structure that fits in the available microcontroller memory, and (iii) an analysis of the resulting accuracy and computational performance on the target and other possible candidate embedded platforms.

This paper is structured as follows: Section 2 begins by introducing some recent works related to the state of the art for gesture recognition. Section 3 describes the dataset used to define and evaluate the performance of the neural network, and the data preprocessing and augmentation techniques used. Section 4 describes the network architecture and its implementation on a microcontroller. Section 5 presents some experiments conducted for model training and validation. Section 6 discusses the obtained results and outlines possible future improvements. Finally, Section 7 draws some conclusions.

## 2. Related Works

Gesture recognition plays a crucial role in various Human–Computer Interaction (HCI) applications, including virtual game control, sign language interpretation, assisted living, and robot control [27]. The hand serves as an effective and essential medium for interaction in these systems. Gestures can be broadly classified into two types: static gestures, which involve fixed hand shapes, and dynamic gestures, which involve a sequence of hand movements, such as waving. Therefore, developing a system that can automatically and accurately recognize or categorize these gestures is essential.

Prior studies on gesture recognition are generally classified into two principal categories based on the input modalities: image-based approaches [28,29] and skeleton-based approaches [30,31]. Image-based methods typically employ Red–Green–Blue (RGB) or RGB–Depth (RGB-D) imagery to extract visual features for gesture recognition. Conversely, skeleton-based methods utilize temporal sequences of two-dimensional (2D) or three-dimensional (3D) hand joint coordinates to model and predict gesture patterns.

Static gestures are characterized by fixed angles between the fingers and remain unchanged over time, such as pointing or grasping. In contrast, dynamic gestures involve changing finger angles over a period of time—for example, waving or drawing letters in the air [32]. Based on this distinction, datasets for static gestures are typically composed of still images, while dynamic gesture datasets require skeletal data or/and temporal data, often captured using inertial sensors that record motion over time. For static gesture recognition, neural networks, especially convolutional neural networks (CNNs) [33,34,35], have proven highly effective due to their capacity to learn spatial features and extract detailed patterns directly from RGB images. More recently, transformer-based models [36] have advanced the field by enabling the capture of temporal and contextual information. This has been particularly beneficial in applications such as sign language recognition and HCI, where understanding the sequence and context of gestures is essential.

Regarding dynamic hand gesture recognition, a brief overview of recent works that use different types of signals follows. We nevertheless underline that all these works focus on the accuracy parameter and not on the inference time and/or memory footprint for a possible implementation of the algorithm directly on a wearable device.

Singh et al. [37] present a deep learning approach for the dynamic recognition of hand gestures using three-dimensional (3D) skeletal data. The proposed model integrates a Bidirectional Long Short-Term Memory (Bi-LSTM) network with a soft attention mechanism. Experimental results demonstrate that the Bi-LSTM effectively captures temporal dependencies, while the subsequent soft attention layer enhances the extraction of salient features. Gesture classification is then performed using a Multi-Layer Perceptron (MLP) followed by a softmax activation function. Additionally, a data augmentation strategy is introduced, which significantly improves the model’s generalization capabilities and mitigates the risk of overfitting.

Liu et al. [38] propose a novel and efficient gesture recognition framework using multi-channel surface electromyography (sEMG) signals acquired from a wearable sensor (Myo). To address the trade-off between accuracy and computational efficiency in existing methods, the model integrates a lightweight convolutional neural network (CNN) with a Lightweight Feature Extraction (LFE) block for effective multi-scale feature aggregation. A pyramid input structure enhances contextual understanding across resolutions, while transfer learning with pre-trained weights improves feature representation and accelerates convergence. Additionally, a Broad Attention Learning (BAL) block is introduced to further refine recognition performance. The proposed approach is validated on a dynamic gesture dataset, demonstrating superior generalization and efficiency.

With reference to accelerometer sensors, Rafiq et al. [39] conducted an experimental evaluation using the SmartWatch Gesture dataset [26] (the same dataset used in this work) and the Motion Gesture dataset. The proposed method resulted in average test accuracies of 57.0%, 64.6%, and 69.3% by using one, three, and five samples for six different gestures. Their method, called Latent Embedding Exploitation, learns novel gesture classes incrementally using a few samples from motor-impaired individuals, but they use a subset of the total classes of the dataset.

Other works that used similar datasets only addressed the problem of recognizing a smaller number of classes, usually just eight [26,40]. In particular, ref. [41] is perhaps the most similar to our work as it also used an LSTM network, but to recognize just eight classes on an ARM Cortex-A9 microprocessor with plenty of available memory, achieving a classification error rate of 11.43%, without using a subject-based split of the training and validation data.

## 3. Materials and Methods

**Figure 1:**
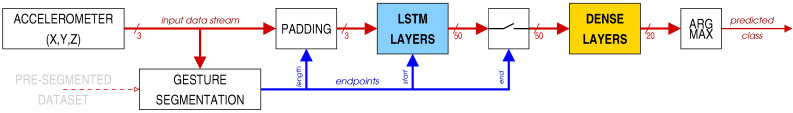
Overview of the stages of the reference recognition framework.

The system we envision processes data acquired at a low sampling frequency (compatible with human motion dynamics) from a 3-axis accelerometer through an RNN to be implemented on a low-power microcontroller suitable for wearable devices. An overview of the data flow can be seen in Figure 1.

The raw data from the accelerometer must be segmented into individual gestures. This task was already manually performed in the dataset we employed, so the recorded segment length is just forwarded to the following stages. In a real-time system, a dedicated gesture segmentation unit will be needed, whose design is beyond the scope of this paper, though it is envisioned it could use an auxiliary output from the LSTM layers trained to signal the endpoint. Short sequences are then padded (by repeating the last available sample) to a fixed length, and passed to the LSTM layers that analyse them. At the end of each sequence, the state of the final LSTM layer is passed on to the dense layers which perform the actual classification. The exact details of each stage are detailed in the following sections.

### 3.1. Dataset

**Figure 2:**
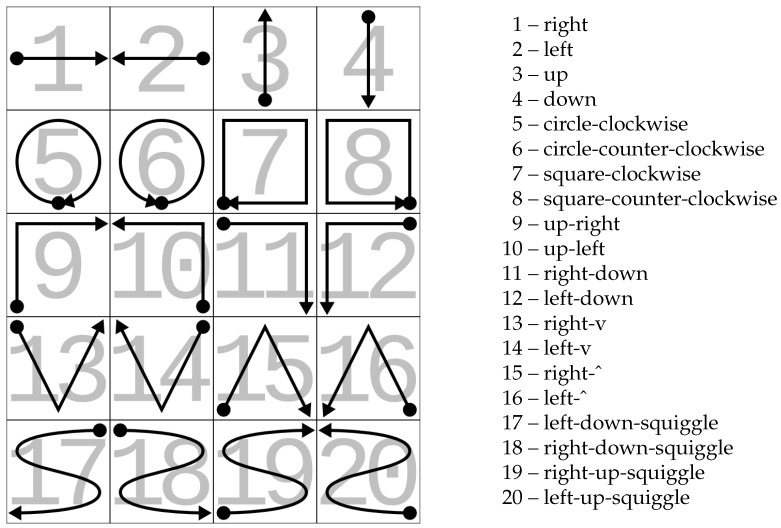
Graphical depiction of the different gestures in the dataset. Source: https://tev.fbk.eu/resources/smartwatch (accessed on 3 April 2025) [26], reproduced under license: “SmartWatch Gestures Dataset is provided for research or academic purposes only”.

The dataset used to define and assess the performance of the neural network consists of acceleration data collected from a wrist-worn smartwatch while the user performed about 20 repetitions of 20 different hand gestures. The collection was repeated by 8 different users, for a total of 3251 sequences of varying length [26]. A graphical representation of the different gestures can be seen in Figure 2.

The data consist of 3-axis acceleration values sampled at about 9.1 Hz (110 ms sampling interval) and their associated timestamp. Actually, three different timestamps are provided, two of which are absolute times (referred to the Epoch as per POSIX specifications), and the other is an internal timer timestamp. Sampling turned out not to be perfectly uniform, probably due to the hardware architecture of the smartwatch employed, which did not synchronize the accelerometer sampling time base with the main clock (which is a very common practice since integrated MEM-based accelerometers are almost always self-clocked). However, after ensuring that the sampling rate jitter was indeed small enough (the standard deviation between samples is about 11 ms, i.e., 10% of the nominal period) to make it negligible for activity recognition purposes, all timestamps had been discarded to avoid leaking information from the data acquisition process into the recognizer.

### 3.2. Data Preprocessing and Augmentation

**Figure 3:**
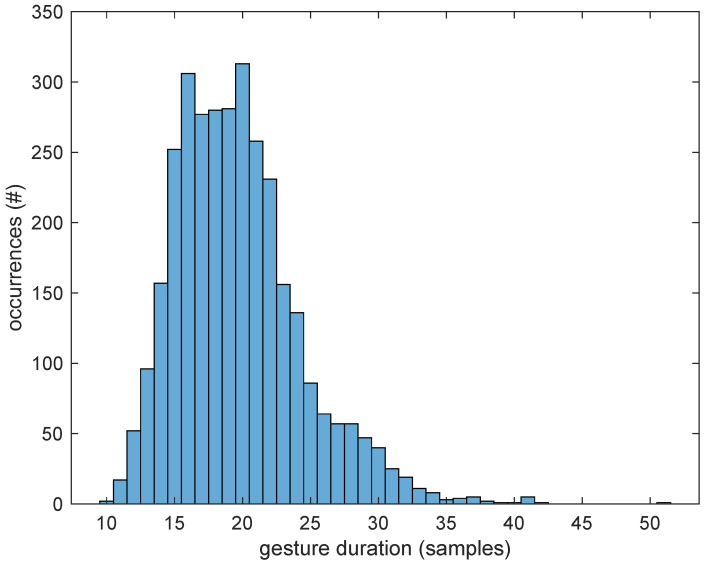
Histogram of the duration of the sequences in the dataset: number of occurrences (#) of the recorded sequence lengths for each possible length expressed in samples.

As mentioned above, the dataset is already segmented into separate gestures. The segmentation was actually performed by the users themselves, who tapped the screen of the smartwatch before and after performing the gesture. But the duration of each segment is obviously different, varying from 10 samples (1.1 s) to 51 samples (5.6 s), as shown in Figure 3.

**Figure 4:**
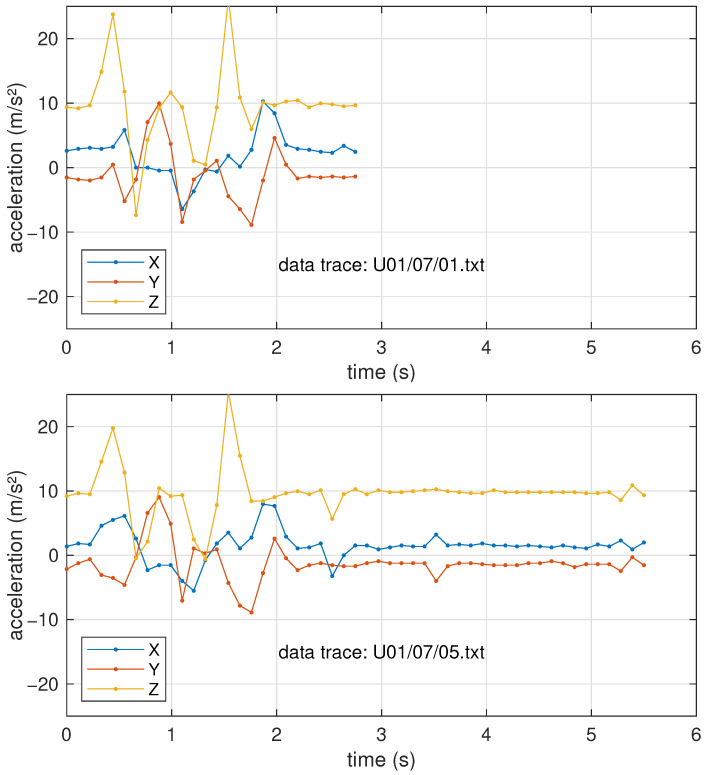
Examples of two recordings for the same movement (gesture “7 – square-clockwise”).

To feed the input layer of the neural network, all sequences need to be the same length. From the histogram in Figure 3, a common duration of 4 s (36 samples) was chosen, as only a few outliers needed to be truncated (and the discarded data turned out to be essentially constant, denoting a delay from the user in tapping the watch to stop the recording). On the contrary, the shorter segments were padded to the same length by repeating the last sample. Since most gestures end with a rest condition, it is reasonable to assume that the last acceleration samples are just due to the gravitational force, which should remain constant if no further movements are performed. An example of a couple of recordings to show this are reported in Figure 4.

On the other hand, simple zero-padding, though it is a very common practice, would mean assuming the movement ended with a free fall, which is not reasonable.

The dataset was then prepared to train and test a network through a 4-fold subject-based cross-validation scheme, whereby a partition of the subjects into 4 groups of 2 is defined, and each group is used as validation for a network trained using the other 3 groups of subjects, as shown in Table 1. This subject-based approach was chosen because it ensures that validation is performed with data recorded from persons never before seen during training, thus giving some confidence that the neural network is general enough to cope with real-world data acquired from diverse people.

The resulting split was already well balanced across the different classes (since most users performed the same number of repetitions of each exercise), as can be seen in Table 2, so no further adjustments were made.

**Figure 5:**
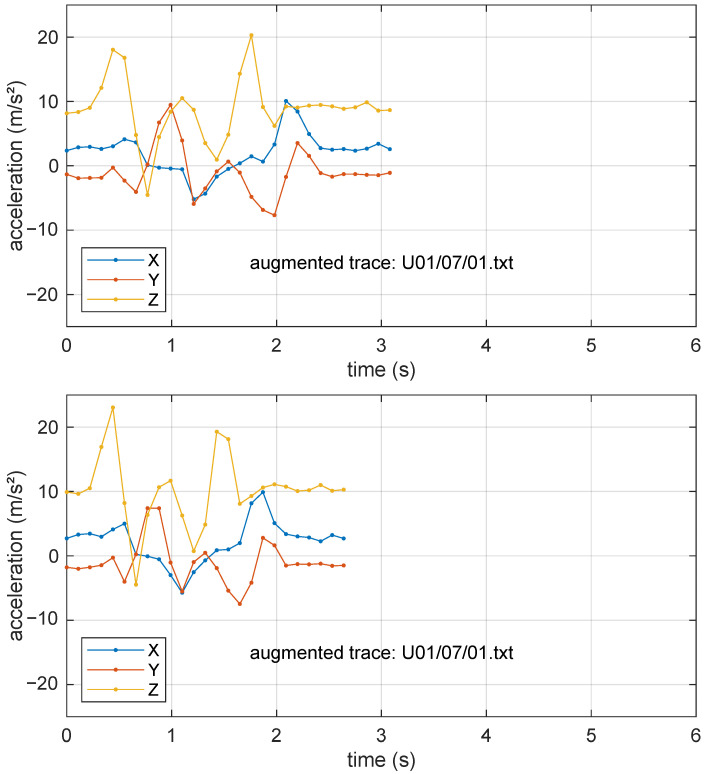
Examples of the effect of data augmentation on the data sequence U01/07/01 shown in Figure 4. The most prominent effects are the random duration and scale alterations.

Finally, each training set was doubled in size using a data augmentation technique that included noise addition, random scaling, and time warping. The noise addition stage adds Gaussian white noise with a standard deviation equal to 5% of the signal standard deviation, while random scaling independently alters the magnitude of the three acceleration channels by a random amount between −10% and +10%. The time warping stage uses linear interpolation to extend or compress the gesture duration by up to 30%. As an example of what these “augmented” traces look like, Figure 5 shows two samples of the sequence U01/07/01 (already shown in its original shape in Figure 4) after being processed with the aforementioned steps.

## 4. Neural Network Architecture

Given the time-domain nature of the signals to be recognized, the neural network was chosen among the family of recurrent networks, or RNNs, so that the information on previous samples and the timing correlation between them could be better exploited. In particular, LSTM networks have been employed so that long-range correlations between samples can also be understood. The nodes of these networks are indeed more complex than regular neurons, and include, besides a hidden state, a forget gate, an update gate, and an output gate. Together, they allow for more trainable parameters while alleviating the problem of gradient vanishing or exploding during the training phase.

In order to take into account the memory limitations of the microcontroller platform on which the network is supposed to run, it is useful to perform an estimation of the computational and memory costs of different design choices.

Consider the *j*-th LSTM layer composed of $N_{j}$ cells attached to $L_{j}$ inputs (with $L_{1}=3$ because of the 3-axis acceleration data being processed, and $L_{j+1}=N_{j}$ for subsequent layers). Each LSTM cell has 4 McCulloch–Pitts neurons inside (3 to compute the “gating” factors with a “sigmoid” activation, and 1 to compute the candidate next state with a “tanh” activation) that act upon both the input ($L_{j}$ values) and the previous state of the entire layer ($N_{j}$ values). Each cell thus needs $4\;\cdot \;(L_{j}+N_{j}+1)$ trainable weights (also counting the bias). The operation of the whole layer can basically be cast as a matrix multiplication between $W_{j}$ and $x_{j}$ followed by the computation of the activation functions, with $W_{j}\in R^{\;4\;N_{j}\times \left(L_{j}+N_{j}+1\right)}$, and $x_{j}\in R^{\;L_{j}+N_{j}+1}$ is the concatenation of the input vector, state vector (previous output), and the constant 1 for the bias. In terms of computational cost, the matrix–vector multiplication requires $4\;\left(L_{j}+N_{j}+1\right)\;N_{j}$ multiply–accumulate operations (MACs). The computation of the non-linear activation functions is a bit more involved as it requires divisions. Counting only the floating point operations, a typical algorithm may require 5 MACs, 6 ADDs, and 1 DIV. For an LSTM layer, $5\;N_{j}$ activations need to be computed and $3\;N_{j}$ MACs are needed to combine them at the gates, leading to a grand total of approximately $4\;\left(L_{j}+N_{j}+17\right)\;N_{j}$ operations (neglecting the fact that the DIV is much more expensive than the others, but so are memory accesses, so this can only be a rough estimate). These layers also need to be evaluated for each sample in the input sequence, that is, 36 times in our case.

Dense layers, on the other hand, are much simpler. They need (using the same notation) $\left(L_{j}+1\right)\;N_{j}$ weights to be stored and exactly one MAC operation per weight. ReLU activations are also much quicker to compute and basically negligible. Most importantly, they need to be evaluated only once per entire sequence.

With this information, it is possible to estimate the complexity in terms of number of weights ($N_{w}$) for, e.g., a four-layer network comprised of two LSTM layers and two dense layers:

(1) $$N_{w}=4\;\left(N_{1}+4\right)\;N_{1}+4\;\left(N_{1}+N_{2}+1\right)\;N_{2}+\left(N_{2}+1\right)\;N_{3}+20\;\left(N_{3}+1\right)$$

where it was assumed $L_{1}=3$ and $N_{4}=20$ as per problem specifications.

**Figure 6:**
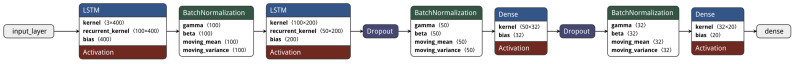
Architecture of the implemented NN.

$N_{1}$, $N_{2}$, and $N_{3}$ represent the design parameters that need to be chosen as a compromise between network complexity and classification performance. After extensive experimentation, the final choice is the structure shown in Figure 6, with $N_{1}=100$, $N_{2}=50$, and $N_{3}=32$, resulting in an estimated number of trainable parameters (not including convergence-aiding layers such as batch normalization) $N_{w}=74,092$, which corresponds to about 30% memory usage for weight storage, and requires about 2,932,692 FLOPs to be computed.

The hidden dense layer uses the ReLU activation function, and the model was fitted with the aid of an L2 kernel regularization function to further prevent overfitting from occurring. The output layer finally uses softmax activation to help predict class membership probabilities. Normalization and dropout stages (with a 30% discard rate) were also added to help with data scaling and alleviating the risk of overfitting. Table 3 shows the details of each layer with the count of trainable parameters. This architecture requires a total of 74,820 parameters, which can easily fit into the available memory of the chosen microcontroller.

### 4.1. Model Training

The network presented previously was trained using the TensorFlow 2.12.0 framework with the aid of Keras 2.12.0 to ease model definition and fitting, using the Google Colaboratory platform. The Adam optimizer with a learning rate of 0.0005 was used. Up to 70 training epochs with a batch size of 32 were scheduled, but the early stopping criterion (with a patience set to 10) ensured that on average only 30 epochs were needed to train each fold.

### 4.2. Microcontroller Platform

The platform chosen for the application was a RushUp Cloud-JAM L4 board (https://github.com/rushup/Cloud-JAM-L4, accessed on 16 April 2025), based on an STMicroelectronics STM32L476RG microcontroller, which features an 80 MHz ARM Cortex-M4 32 bit CPU + FPU and integrates 1 MiB of flash memory and 128 KiB of SRAM. It draws 3 mA of current at full speed. The board is also equipped with an STMicroelectronics iNEMO LSM6DSL inertial platform, and, among other sensors and stuff, a WiFi module, making it ideal for acceleration-based gesture recognition tasks such as the one presented in this paper.

The trained model of the neural network was converted to C code suitable to be run on the microcontroller with the aid of the STM32Cube AI tool (version 10.0) (https://www.st.com/en/embedded-software/x-cube-ai.html, accessed on 16 April 2025), which is integrated into the STM32Cube IDE. This tool provides a comprehensive solution for importing Keras (or other) models, analyzing them for compatibility and memory usage, and converting them into optimized C code tailored for the target architecture. The resulting network can be evaluated with test input data both on the host computer and directly on the device, enabling the collection of various performance metrics such as execution time, hardware operation counts, and accuracy.

## 5. Results

The network so trained had been tested both on the Colab platform and directly on the microcontroller platform, to ensure model validity and that the porting on the microcontroller did not introduce unwanted discrepancies. The results turned out to be indeed identical.

**Figure 7:**
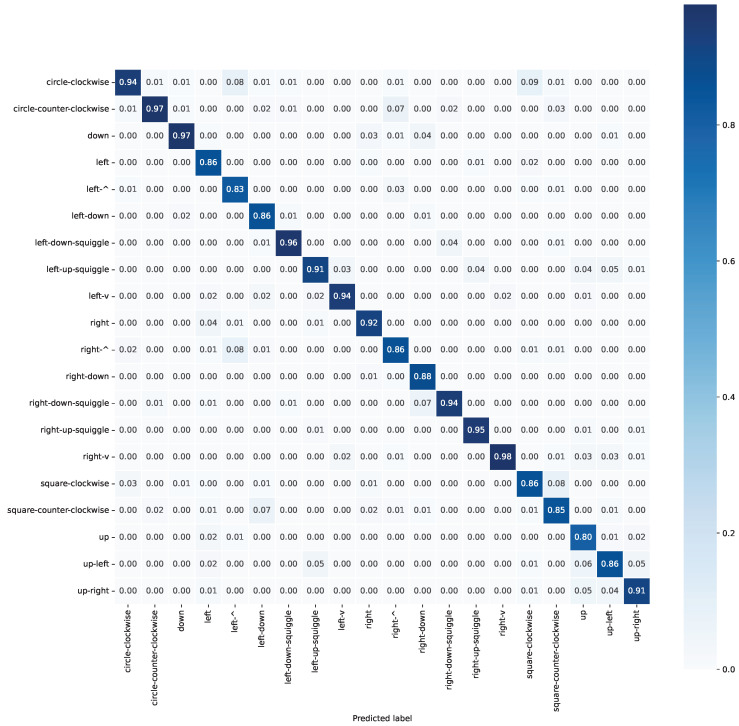
Average confusion matrix of the 4 folds.

The resulting confusion matrix, averaged across all four folds, is reported in Figure 7.

Specifically, each fold resulted in the performance shown in Table 4, with an overall accuracy (averaged over all four folds) of 90.25%.

The addition of noise performed during the data augmentation phase should theoretically help improve noise immunity during inference. To test the network robustness to noise, an additional experiment in which white Gaussian noise was artificially added to the validation set before inference was performed. It is hard to exactly quantify the effect, as training and noise are inherently non-deterministic processes so the results tend to vary slightly from run to run, but we consistently obtained an accuracy of 90% ± 0.5% in all our test runs with a noise standard deviation of 0.2 m/s^2^. This level of noise was chosen because it mimics the random fluctuations that can be seen, e.g., in the tail portion of Figure 4, and also corresponds (on average) to the 5% that was added during data augmentation.

**Figure 8:**
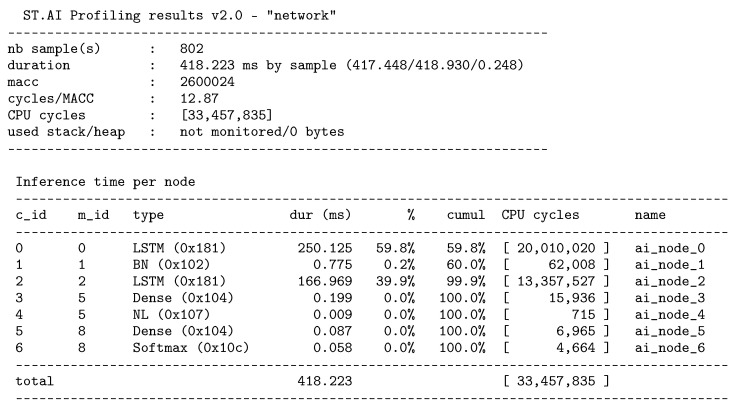
Report on the microcontroller implementation.

With regard to the porting of the neural network on the embedded platform, the porting consumed 291.96 KiB of flash memory for weight storage and 17.22 KiB of RAM for the activations. Finally, a summary of the computational complexity and the ensuing computation times are reported in Figure 8.

The reference platform used in this paper (Cloud-JAM L4) was chosen for its low-power capabilities, as it employs an L-series microcontroller. As a reference, the network performance was also evaluated on higher-end microcontroller platforms using the ST Edge AI Developer Cloud (https://stm32ai.st.com/st-edge-ai-developer-cloud/, accessed on 9 May 2025), a free online service for developing/testing AI models on ST devices, including MCU, MPU, and MEMS sensors, which offers tools for creation, optimization, and benchmarking of these models. Table 5 shows the results achieved in terms of inference time, computational complexity, specifically MAC instructions, and memory footprint to run the model deployed in different ARM Cortex-M MCUs. The model’s weights were represented in 32-bit floating point precision.

## 6. Discussion

The proposed network architecture allowed a compact implementation on the selected microcontroller, using only 28.5% of the available flash memory, leaving plenty of space for application code and other system functionalities, while achieving a 90.25% average accuracy on a 4-fold subject-based cross-validation test. In terms of computation speed, the model runs in less than half a second, while processing 4 s worth of input data, despite the platform we used being the one with the lowest clock frequency amongst those customarily used for edge AI. This means that the system can easily run in real time, actually eight times faster, allowing for a clock frequency reduction (if the rest of the application allows) to further reduce power consumption from the nominal 3 mA at full speed.

As far as accuracy, the results seem very promising, as mistakes were basically only made between a gesture and another which was either a superset or a subset of the movements, or a “smoothed” version of the other, such as 9% of “square-clockwise” movements were incorrectly labeled as “circle-clockwise”, as can be seen in the confusion matrix shown in Figure 7. Future research can try and address this problem by adding a better gesture segmentation algorithm and/or adding additional features, such as gesture duration, to the training data. Moreover, the network proved to be quite immune to noise, as tests performed by adding white Gaussian noise to the inference data with a power similar to that measured on real samples did not show significant differences in the overall accuracy.

As far as the positioning of the achieved performance with respect to the existing literature, there are no published results for a full 20-class recognition accuracy on this dataset. Some other works that used the same dataset are listed in Table 6. These include [39], which used the full dataset to pre-train a network then tested in a continual learning setting (named LEE, Latent Embedding Exploration) on a smaller number of classes (6), achieving accuracies between 57.0% and 69.3% depending on the length of the used sequence. Ref. [41] also used an LSTM network to recognize eight classes (namely gestures 1, 2, 3, 4, 13, 16, 17, and 19 from Figure 2), achieving a classification error rate of 11.43%, which is comparable to our accuracy, but using a much more powerful embedded platform based on an ARM Cortex-A9 microprocessor with plenty of available memory, and without performing a subject-based split of the training data. The same authors of the dataset used in this work presented some classification results on a slightly different and smaller version of it [40], where they achieved an accuracy up to 95.8% using an SVM-based (which in general is much more computationally demanding than a neural network) classifier, but again without performing a subject-based split of the training data. Using a leave-one-subject-out validation, which is more similar to our 4-fold cross-validation, resulted in an accuracy ranging between 91.08% and 92.33% as shown in the table. It is worth noting that, to the best of our knowledge, there are no works in the published literature that tried to recognize this dataset on an embedded microcontroller platform, as shown in the aforementioned Table 6.

**Table 6:** Comparative studies with state-of-the-art gesture recognition methodologies on the SmartWatch Gesture Dataset.

Work	Method	Parameter Memory	No. Classes	Accuracy	Validation Method	Embedded Implementation
Rafiq et al. [39]	LEE	N/A	6	69.30%	holdout	no
Shin et al. [41]	LSTM-RNN	17.25 KB	8	88.57%	holdout	no
Porzi et al. [40]	fGAK-SVMHAAR-SVM	N/A	8	92.33% 91.08%	k-fold	no
This Work	LSTM	292.27 KiB	20	90.25%	k-fold	yes

## 7. Conclusions

In this work, we presented a neural network for the dynamic hand gesture recognition task, using accelerometer data acquired from a smartwatch. It was implemented on a low-power microcontroller (from the STM32L4 series), achieving much-better-than-real-time computation speed and an overall accuracy above 90% over all the 20 classes examined. This accuracy compares favorably with the state of the art for this dataset, as other works that attained marginally better results only used a small subset of the classes, and none actually implemented the network on a resource-constrained embedded platform. Indeed, the proposed architecture uses only about one quarter of the available storage space for weights, thus posing to become a viable platform for the development of wearable smart sensors.

## Figures and Tables

**Table 1:** Detail of the 4-fold train-validation split scheme of the 8 users.

Fold	Train	Validation
#1	U01, — , U03, U04, U05, — , U07, U08	— , U02, — , — , — , U06, — , —
#2	— , U02, U03, U04, U05, U06, U07, —	U01, — , — , — , — , — , — , U08
#3	U01, U02, — , U04, — , U06, U07, U08	— , — , U03, — , U05, — , — , —
#4	U01, U02, U03, — , U05, U06, — , U08	— , — , — , U04, — , — , U07, —

**Table 2:** Consistency of the dataset: for each fold, the number of sequences used for validation and training is reported.

Gesture	Fold #1		Fold #2		Fold #3		Fold #4	
1 – right	40	120	40	120	40	120	40	120
2 – left	40	121	40	121	41	120	40	121
3 – up	43	123	40	126	43	123	40	126
4 – down	46	120	40	126	40	126	40	126
5 – circle-clockwise	40	120	40	120	40	120	40	120
6 – circle-counter-clockwise	40	124	41	123	43	121	40	124
7 – square-clockwise	40	121	40	121	41	120	40	121
8 – square-counter-clockwise	42	122	40	124	42	122	40	124
9 – up-right	40	121	40	121	41	120	40	121
10 – up-left	40	124	40	124	44	120	40	124
11 – right-down	40	122	40	122	42	120	40	122
12 – left-down	40	121	40	121	41	120	40	121
13 – right-v	40	121	40	121	41	120	40	121
14 – left-v	45	123	40	128	43	125	40	128
15 – right-^	40	122	40	122	42	120	40	122
16 – left-^	40	121	40	121	41	120	40	121
17 – left-down-squiggle	41	122	40	123	42	121	40	123
18 – right-down-squiggle	41	122	41	122	41	122	40	123
19 – right-up-squiggle	40	122	40	122	42	120	40	122
20 – left-up-squiggle	40	121	40	121	41	120	40	121

**Table 3:** Summary of the individual layers in the proposed NN architecture.

Layer Type	Output Shape	Parameters
LSTM	(None, 36, 100)	41,600
BatchNormalization	(None, 36, 100)	400
LSTM	(None, 50)	30,200
Dropout	(None, 50)	0
BatchNormalization	(None, 50)	200
Dense	(None, 32)	1632
Dropout	(None, 32)	0
BatchNormalization	(None, 32)	128
Dense	(None, 20)	660

Total parameters: 74,820 (292.27 KiB), of which 74,456 (290.84 KiB) are trainable.

**Table 4:** Performance summary of the network architecture over all 4 folds.

Fold	Precision	Recall	F1 Score	Accuracy
#1	0.90	0.89	0.88	0.89
#2	0.94	0.93	0.93	0.93
#3	0.92	0.90	0.90	0.90
#4	0.90	0.89	0.89	0.89

**Table 5:** Results achieved on the MCU memory footprint and inference time, measured with ST Edge AI Developer Cloud.

Board Name	Clock Frequency (MHz)	MCU	Inference Time (ms)	CPU Cycles
STM32H7S78-DK	600	Arm Cortex-M7	51.03	30,622,885
STM32H735G-DK	500	Arm Cortex-M7	36.93	20,311,684
NUCLEO-H743ZI2	480	Arm Cortex-M7	43.91	21,078,404
STM32H573I-DK	250	Arm Cortex-M33	108.4	27,110,237
STM32F469I-DISCO	180	Arm Cortex-M4	168.8	30,378,125
NUCLEO-F401RE	84	Arm Cortex-M4	316.2	26,561,411
Cloud-JAM L4 (this work)	80	Arm Cortex-M4	418.2	33,457,835

## Data Availability

The original contributions presented in this study are included in the article. Further inquiries can be directed to the corresponding author.

## References

[1] Biagetti G., Crippa P., Falaschetti L., Orcioni S., Turchetti C. (2018). Human activity monitoring system based on wearable sEMG and accelerometer wireless sensor nodes. Biomed. Eng. Online.

[2] Hochreiter S., Schmidhuber J. (1997). Long short-term memory. Neural Comput..

[3] Falaschetti L., Alessandrini M., Biagetti G., Crippa P., Turchetti C. (2022). ECG-Based Arrhythmia Classification using Recurrent Neural Networks in Embedded Systems. Procedia Comput. Sci..

[4] Musci M., De Martini D., Blago N., Facchinetti T., Piastra M. (2021). Online Fall Detection Using Recurrent Neural Networks on Smart Wearable Devices. IEEE Trans. Emerg. Top. Comput..

[5] Alessandrini M., Biagetti G., Crippa P., Falaschetti L., Turchetti C. (2021). Recurrent Neural Network for Human Activity Recognition in Embedded Systems Using PPG and Accelerometer Data. Electronics.

[6] Castells-Rufas D., Borrego-Carazo J., Carrabina J., Naqui J., Biempica E. (2022). Continuous touch gesture recognition based on RNNs for capacitive proximity sensors. Pers. Ubiquitous Comput..

[7] Falaschetti L., Biagetti G., Crippa P., Alessandrini M., Giacomo D.F., Turchetti C. (2022). A Lightweight and Accurate RNN in Wearable Embedded Systems for Human Activity Recognition. Smart Innov. Syst. Technol..

[8] Gaud N., Rathore M., Suman U. (2024). MHCNLS-HAR: Multiheaded CNN-LSTM-Based Human Activity Recognition Leveraging a Novel Wearable Edge Device for Elderly Health Care. IEEE Sens. J..

[9] Saddaoui R., Gana M., Hamiche H., Laghrouche M. (2024). Wireless Tag Sensor Network for Apnea Detection and Posture Recognition Using LSTM. IEEE Embed. Syst. Lett..

[10] Yu X., Qiu H., Xiong S. (2020). A Novel Hybrid Deep Neural Network to Predict Pre-impact Fall for Older People Based on Wearable Inertial Sensors. Front. Bioeng. Biotechnol..

[11] Abadi M., Agarwal A., Barham P., Brevdo E., Chen Z., Citro C., Corrado G.S., Davis A., Dean J., Devin M. (2015). TensorFlow: Large-Scale Machine Learning on Heterogeneous Systems. https://zenodo.org/records/15009305.

[12] STMicroelectronics (2021). X-CUBE-AI—AI Expansion Pack for STM32CubeMX—STMicroelectronics. https://www.st.com/en/embedded-software/x-cube-ai.html.

[13] Sinkevych O., Boyko Y., Rechynskyi O., Sokolovskii B., Monastyrskii L. Embedding Sequence Model in STM32 Based Neuro-Controller. Proceedings of the 2021 IEEE 12th International Conference on Electronics and Information Technologies (ELIT).

[14] Biagetti G., Crippa P., Falaschetti L., Tanoni G., Turchetti C. (2018). A comparative study of machine learning algorithms for physiological signal classification. Procedia Comput. Sci..

[15] Wei B., Zhang S., Diao X., Xu Q., Gao Y., Alshurafa N. (2023). An End-to-End Energy-Efficient Approach for Intake Detection With Low Inference Time Using Wrist-Worn Sensor. IEEE J. Biomed. Health Inform..

[16] Kumari N., Yadagani A., Behera B., Semwal V.B., Mohanty S. (2024). Human motion activity recognition and pattern analysis using compressed deep neural networks. Comput. Methods Biomech. Biomed. Eng. Imaging Vis..

[17] Begum H., Chowdhury O., Hridoy M.S.R., Islam M.M. (2024). AI-Based Sensory Glove System to Recognize Bengali Sign Language (BaSL). IEEE Access.

[18] Sharma V., Sharma A., Saini S. (2024). Real-time attention-based embedded LSTM for dynamic sign language recognition on edge devices. J. Real-Time Image Process..

[19] DelPreto J., Hughes J., D’Aria M., de Fazio M., Rus D. (2022). A Wearable Smart Glove and Its Application of Pose and Gesture Detection to Sign Language Classification. IEEE Robot. Autom. Lett..

[20] Makaussov O., Krassavin M., Zhabinets M., Fazli S. A Low-Cost, IMU-Based Real-Time On Device Gesture Recognition Glove. Proceedings of the 2020 IEEE International Conference on Systems, Man, and Cybernetics (SMC).

[21] Nguyen-Trong K., Vu H.N., Trung N.N., Pham C. (2021). Gesture Recognition Using Wearable Sensors With Bi-Long Short-Term Memory Convolutional Neural Networks. IEEE Sens. J..

[22] Biagetti G., Crippa P., Falaschetti L., Focante E., Martínez Madrid N., Seepold R. (2020). Machine Learning and Data Fusion Techniques Applied to Physical Activity Classification Using Photoplethysmographic and Accelerometric Signals. Procedia Comput. Sci..

[23] Chevalier G. (2016). LSTMs for humAn Activity Recognition. https://github.com/guillaume-chevalier/LSTM-Human-Activity-Recognition.

[24] Aklah Z.T., Hassan H.T., Al-Safi A., Aljabery K. (2024). Fall Detection in Q-eBall: Enhancing Gameplay Through Sensor-Based Solutions. J. Sens. Actuator Netw..

[25] Coffen B., Mahmud M. TinyDL: Edge Computing and Deep Learning Based Real-time Hand Gesture Recognition Using Wearable Sensor. Proceedings of the 2020 IEEE International Conference on E-health Networking, Application & Services (HEALTHCOM).

[26] Costante G., Porzi L., Lanz O., Valigi P., Ricci E. Personalizing a smartwatch-based gesture interface with transfer learning. Proceedings of the 2014 22nd European Signal Processing Conference (EUSIPCO).

[27] De Smedt Q., Wannous H., Vandeborre J.P. (2019). Heterogeneous hand gesture recognition using 3D dynamic skeletal data. Comput. Vis. Image Underst..

[28] Wang C., Liu Z., Chan S.C. (2015). Superpixel-Based Hand Gesture Recognition With Kinect Depth Camera. IEEE Trans. Multimed..

[29] Molchanov P., Gupta S., Kim K., Kautz J. Hand gesture recognition with 3D convolutional neural networks. Proceedings of the 2015 IEEE Conference on Computer Vision and Pattern Recognition Workshops (CVPRW).

[30] Chen X., Guo H., Wang G., Zhang L. Motion feature augmented recurrent neural network for skeleton-based dynamic hand gesture recognition. Proceedings of the 2017 IEEE International Conference on Image Processing (ICIP), IEEE.

[31] Núñez J.C., Cabido R., Pantrigo J.J., Montemayor A.S., Vélez J.F. (2018). Convolutional neural networks and long short-term memory for skeleton-based human activity and hand gesture recognition. Pattern Recognit..

[32] Reifinger S., Wallhoff F., Ablassmeier M., Poitschke T., Rigoll G. (2007). Static and dynamic hand-gesture recognition for augmented reality applications. Proceedings of the Human-Computer Interaction, HCI Intelligent Multimodal Interaction Environments: 12th International Conference, HCI International 2007.

[33] Chavan S., Yu X., Saniie J. Convolutional neural network hand gesture recognition for American sign language. Proceedings of the 2021 IEEE International Conference on Electro Information Technology (EIT).

[34] Singh A., Hashmi F.E., Tyagi N., Jayswal A.K. Impact of colour image and skeleton plotting on sign language recognition using convolutional neural networks (CNN). Proceedings of the 2024 14th International Conference on Cloud Computing, Data Science & Engineering (Confluence).

[35] Wang Y., Sun W., Rao R. (2024). Accurate and Real-time Variant Hand Pose Estimation Based on Gray Code Bounding Box Representation. IEEE Sens. J..

[36] Zhang P., Kong D. Handformer2T: A Lightweight Regression-based Model for Interacting Hands Pose Estimation from A Single RGB Image. Proceedings of the 2024 IEEE/CVF Winter Conference on Applications of Computer Vision (WACV).

[37] Singh R.P., Singh L.D. (2025). Dyhand: Dynamic hand gesture recognition using BiLSTM and soft attention methods. Vis. Comput..

[38] Liu Y., Li X., Yang L. (2025). A wearable sensor-based dynamic gesture recognition model via broad attention learning. Signal Image Video Process..

[39] Rafiq R.B., Shi W., Albert M.V. (2024). Wearable sensor-based few-shot continual learning on hand gestures for motor-impaired individuals via latent embedding exploitation. arXiv.

[40] Porzi L., Messelodi S., Modena C.M., Ricci E. A smart watch-based gesture recognition system for assisting people with visual impairments. Proceedings of the 3rd ACM International Workshop on Interactive Multimedia on Mobile & Portable Devices (IMMPD ’13).

[41] Shin S., Sung W. Dynamic hand gesture recognition for wearable devices with low complexity recurrent neural networks. Proceedings of the 2016 IEEE International Symposium on Circuits and Systems (ISCAS).

